# Baby Steps: Using Intervention Mapping to Develop a Sustainable Perinatal Physical Activity Healthcare Intervention

**DOI:** 10.3390/ijerph18115869

**Published:** 2021-05-30

**Authors:** Anna M. Dieberger, Mireille N. M. van Poppel, Estelle D. Watson

**Affiliations:** 1 Department of Obstetrics and Gynaecology, Medical University of Graz, Auenbruggerplatz 14, 8036 Graz, Austria; 2 Institute of Human Movement Science, Sport and Health, University of Graz, Mozartgasse 14, 8010 Graz, Austria; mireille.van-poppel@uni-graz.at; 3 Centre for Exercise Science and Sports Medicine, School of Therapeutic Sciences, Faculty of Health Sciences, University of the Witwatersrand, 27 St. Andrews Road, Parktown, Johannesburg 2193, South Africa; estelle.watson@auckland.ac.nz; 4 Department of Exercise Sciences, Faculty of Science, The University of Auckland, Building 907, Suiter Street, Newmarket, Auckland 1142, New Zealand

**Keywords:** physical activity, pregnancy, intervention mapping, theoretical domains framework, behavior change techniques

## Abstract

While the benefits of physical activity (PA) during and after pregnancy have been established, many women do not reach the recommended PA levels during this time. A major barrier found in the literature is a lack of counselling by healthcare providers (HCPs), which is partly caused by the limited knowledge on the topic. The aim of this study was to develop an intervention to improve the promotion of PA by HCPs. We used Intervention Mapping (IM), a theory-based framework to develop an intervention, called “Baby steps”, in a high-income (Austria) and a low-to-middle-income country (South Africa). We applied the following IM steps: (1) A needs assessment to determine the barriers and enablers of PA promotion by HCPs, including a scoping literature review and community needs assessments (qualitative interviews, questionnaires, and focus groups with midwives, obstetricians, and community health workers) to determine the desired outcomes of the intervention. (2) Performance and change objectives were formulated, describing the behaviors that need to change for the intervention to succeed. (3) Based on these objectives, theory-based behavior change techniques were selected, and practical applications were developed. (4) The applications were combined into two evidence-based interventions tailored to each country’s needs. Step (5) and (6) consist of an implementation and evaluation plan, respectively. The intervention is aimed at HCPs, such as midwives and community health workers, consisting of a two-day training course, including practical resources. Combining didactic and interactive education, it addresses both PA knowledge and the skills needed to transfer knowledge and facilitate behavior change. In the future, the intervention’s effect on women’s activity levels during and after pregnancy needs to be studied.

## 1. Introduction

There is a wealth of evidence for the beneficial effects of physical activity (PA) during the prenatal period for both mothers and offspring. Research has shown that being active during pregnancy is safe [1,2,3] and reduces the risk of gestational diabetes mellitus [4], as well as gestational hypertension and preeclampsia [5]. PA can also play an important role in reducing weight gain during pregnancy and in the postpartum period [6]. On the back of this evidence, women with uncomplicated pregnancies are recommended to participate in 150 min of moderate physical activity per week during pregnancy [7,8] and as soon as medically safe postpartum [9].

However, despite the numerous benefits and reputable recommendations, as much as 20–56% of pregnant women are not sufficiently active [10,11,12,13,14]. Regardless of the population and healthcare diversity in high- and low-to-middle income countries, it appears that both struggle with the same issues of prenatal physical inactivity. For example, formative work in South Africa (SA) has shown a decline in PA levels during pregnancy [15], as well as a lot of time spent in sedentary behavior [16]. This is similar to findings in Austria as part of the European DALI study [17]. Additionally, most women do not reach their pre-pregnancy levels of PA after giving birth [18,19].

While barriers such as an unsafe environment and cultural beliefs and norms might differ between low-to-middle and high-income countries, many personal barriers to achieving sufficient PA levels, such as a lack of time and motivation and physical discomforts, appear to be a common challenge [16,20,21,22,23]. In addition, a major barrier found both in our own formative work in SA [24] and in many other studies, is the lack of clear, specific information provided regarding PA during pregnancy by healthcare providers (HCPs) [20,21,25,26]. Indeed, studies have shown that while HCPs were identified as an important source of information [20,27], only a few discuss the topic of PA in pregnancy. At the HCP level, several barriers have been identified to explain this lack of promotion of PA, such as a lack of time and resources and insufficient skills [28].

Therefore, the development of an intervention focusing on improving PA promotion in HCPs is warranted. To develop the intervention, the Intervention Mapping (IM) process was applied. This provides a systematic protocol, which facilitates the development of interventions through an iterative, theory-based process, starting with the identification of health problems and leading to the development, implementation, and evaluation of an intervention. Due to the universal lack of PA promotion by HCPs and the global problem of inactivity during and after pregnancy, the intervention is developed in both a high-income (Austria) and a low-to-middle income country (SA) in order to expand the reach of the intervention and provide possible solutions that could be adapted and implemented across a variety of such settings.

In summary, the aim of this study is to develop a sustainable, evidence-based HCP intervention, focusing on reducing the barriers to PA promotion, and to ultimately improve women’s PA levels during pregnancy and the postpartum period.

## 2. Materials and Methods

### 2.1. Study Setting

The work in this paper was carried out at two sites: (1) Soweto, Johannesburg, SA, which is a previously disadvantaged area and is home to approximately 1.2 million people; and (2) Graz, Austria, which is the capital city of the Southern Austrian province of Styria and Austria’s second largest city, with approximately 330,000 inhabitants.

### 2.2. Intervention Development Group

The main group developing the intervention consisted of a physical activity researcher (E.W.), an epidemiologist with expertise in physical activity interventions and public health (M.V.P.), and a research midwife (A.D.).

### 2.3. Feedback Panel

External stakeholders and researchers with expertise in intervention development, behavior change, motivational interviewing, and PA promotion were consulted for input and feedback: the head of a midwifery school, two professors of midwifery, a professor in exercise science, a human movement scientist, and a research psychologist. Additionally, intervention target group participants, such as midwives and community health workers, have been consulted.

### 2.4. Intervention Mapping

**Figure 1:**
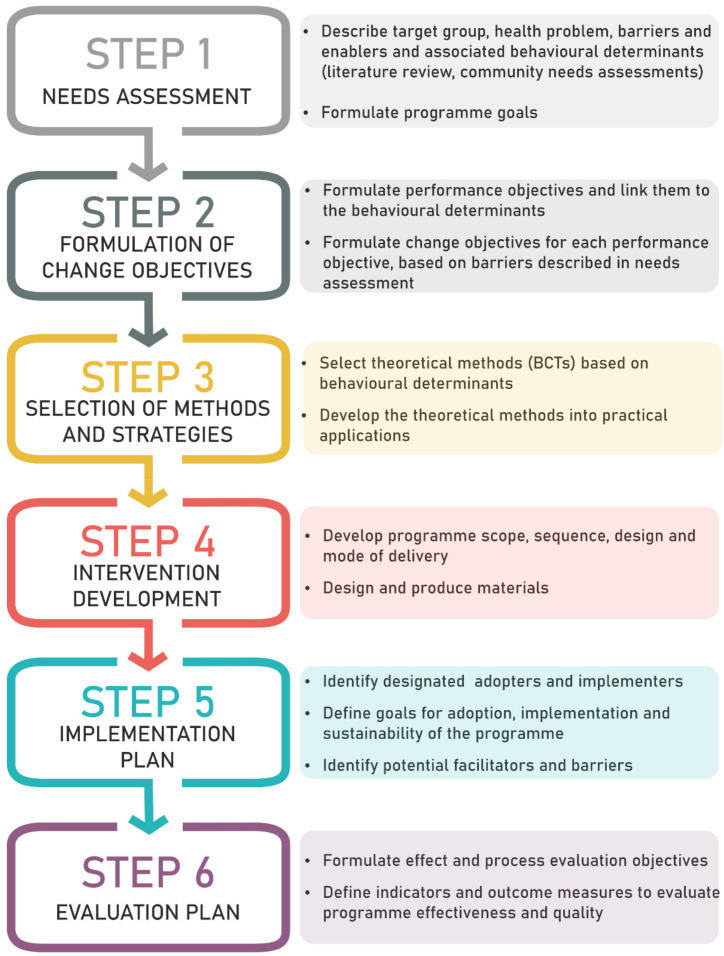
Summary of the six Intervention Mapping steps. BCTs: behavior change techniques.

This paper follows the systematic Intervention Mapping (IM) approach, a framework for the development of behavior change programs that incorporate both evidence and theory into the planning [29,30]. Interventions based on theory are more effective than those with little or no use of theory [31]. IM follows six steps: (1) needs assessment, (2) formulation of change objectives, (3) selection of methods and strategies, (4) intervention development, (5) implementation plan, and (6) evaluation plan [32], which are summarized in Figure 1. While the IM steps were performed for both countries simultaneously, the final developed intervention differs for both countries, reflecting differences in needs and healthcare systems. All intervention details are reported according to the TIDieR [33] checklist and guide (Supplementary File S1).

#### 2.4.1. Step 1: Needs Assessment

The needs assessment uses a combination of published literature and our own formative work to describe the target group, the health problem, and its associated behavioral determinants. A literature review was performed to describe modifiable barriers and enablers of HCPs for the promotion of PA. Additionally, community needs assessments were performed both in Austria and in SA.

In Austria, no previous research on the barriers or enablers of PA promotion around pregnancy in the context of the Austrian healthcare system is available. Therefore, semi-structured interviews were undertaken (*n* = 10) with midwives and obstetricians, as well as the head of a midwifery school, to elicit information on how they currently promote PA to identify barriers and enablers and establish the participants’ attitudes towards a possible intervention.

In SA, previous research among healthcare professionals has already identified potential barriers and enablers of PA promotion during pregnancy [34]. Therefore, in order to provide an effective and sustainable solution, this work focused on the role of community health workers (CHWs) to deliver the intervention. A similar, self-administered questionnaire (Supplementary File S2) was administered to CHWs (*n* = 159) in order to assess their knowledge and attitudes towards general and prenatal PA. Additionally, ten focus groups were conducted on their role in the community, as well as issues of current knowledge and training in PA, to discover potential barriers that might not have been covered by the questionnaire. All methods were employed in accordance with the relevant guidelines and regulations. Informed consent was obtained from all participants. Ethical approval was obtained from the Human Research Ethics Committee of the University of the Witwatersrand (Clearance number M170273).

The updated version of the Theoretical Domains Framework (TDF) [35] was used to sort the findings from the needs assessment according to 14 TDF domains. The TDF domains were used to facilitate the combination of behavioral determinants with behavior change techniques in later steps. In the results section, only TDF domains, for which relevant associated behaviors were found in the needs assessment, are presented.

Step 1 concludes with the formulation of the program goals, defining the overall desired outcomes of the intervention, based on the findings of the needs assessment.

#### 2.4.2. Step 2: Formulation of Change Objectives

In step 2, we determined *performance objectives,* meaning the behavior changes that are necessary to achieve the program goals. The performance objectives were then linked to the behavioral determinants described during the needs assessment. For each performance objective, *change objectives* were generated based on the barriers described in step 1. Thus, change objectives determine the very specific behaviors that will lead to changes in the behavioral determinants, which need to be changed successfully in order to achieve each respective performance objective. The performance and change objectives were refined and finalized after being reviewed by the feedback panel.

#### 2.4.3. Step 3: Selection of Methods and Strategies

For step 3, appropriate theoretical methods to induce the desired behavior changes were chosen, and strategies were formulated on how these methods could be translated into practical applications. As theoretical methods, we selected specific behavior change techniques (BCT) from version 1 of the Behavior Change Technique Taxonomy (BCTTv1) [36]. A BCT is the “active ingredient” of an intervention—an observable and replicable component designed to change the behavior in question.

To help select appropriate BCTs, a recent publication by Johnston et al. was consulted, which aims to identify links between BCTs and their impact on determinants of behavior, based on literature and expert consensus [37]. Therefore, the BCTs for this intervention were selected based on these links, the input from the feedback panel, and taking into consideration their feasibility, relevance, and acceptability for each site.

#### 2.4.4. Step 4: Intervention Development

In this step, the chosen practical applications of the BCTs, which were developed in step 3, were structured into two separate programs, tailored to the specific situations in Austria and SA. In addition to the program scope and sequence, the design and mode of delivery of the program were decided. Prototypes of all needed program materials were designed and pre-tested. To make sure that the program format is acceptable, and the produced materials are culturally appropriate and relevant, the two programs were matched visually, in language and experience, to the intended participant groups in SA and Austria, and future potential participants and stakeholders were asked for input and feedback.

#### 2.4.5. Step 5: Implementation Plan

In step 5, an adoption and implementation plan was developed for the program. First, designated adopters and implementers of the program were identified. Next, program goals were specified for the adoption, implementation (encompassing fidelity, completeness, and dose), and sustainability of the intervention. To achieve these goals, potential facilitators and barriers were determined through brainstorming and a literature search, and methods and practical applications were chosen to address these for each country.

#### 2.4.6. Step 6: Evaluation Plan

Finally, effect and process evaluation objectives were produced, and indicators and outcome measures to evaluate the effectiveness and quality of the intervention, were determined. This was based on the results of the needs assessment in step 1 and summarized into an evaluation plan.

## 3. Results

### 3.1. Step 1: Needs Assessment

#### 3.1.1. Description of the Intervention Target Group

In Austria, the majority of prenatal care is supplied by obstetricians, including five compulsory prenatal check-ups, which are part of the “Mother-Child-Program” [38]. Additionally, a one-hour consult with a midwife at around 18–22 weeks of gestation is offered, which is also part of the standard prenatal care and covered by health insurance. Next to topics like birth preparation and breastfeeding, the subject of health-promoting behaviors, such as nutrition and PA, should be addressed during this consult [38]. As part of standard care after birth, all women are also entitled to home visits by a midwife up to 8 weeks postpartum. This puts midwives in an excellent position to promote physical activity, both in pregnancy and the postpartum period.

In SA, with its healthcare restraints and limited resources, community health workers (CHWs) are a sustainable alternative to healthcare professionals for delivering health promotion. CHWs are essentially members of a community that are supported by the health care system to provide culturally relevant health care services. They typically have no professional training but may have varying degrees of in-service training [39]. In recent years, there has been a drive within SA to re-design the healthcare delivery system to focus on the prevention, health promotion, and advocacy of healthy lifestyles [40]. CHWs play an integral role in this new healthcare model and have already been shown to be effective in improving maternal and child health outcomes [41]. CHWs will see pregnant or postpartum mothers not only in the clinic, but also for follow-up home visits as well, creating opportunities to support families, provide health information, and solve health problems and social challenges [40]. This would be a suitable platform for providing prenatal and postpartum lifestyle advice on PA levels and recommendations. In addition, women appear to prefer receiving health promoting messages from CHWs, as opposed to nurses or midwives at the clinic, which they find unfriendly, intimidating, and unapproachable for questions [24,42].

#### 3.1.2. Description of the Health Problem

Despite the undisputed benefits of PA, many women do not meet the recommended levels during pregnancy and after birth. A lack of advice and information, or conflicting information, is a common barrier to being active in pregnancy and postpartum [20,21,24,26,43,44,45,46,47,48].

In line with this, there have been several successful interventions aimed at increasing physical activity levels, including counselling and education on the topic [49]. Providing information is an effective technique to change PA behavior during pregnancy [50]. Healthcare providers are an important source of information and advice [20,27] and are recommended to discuss physical activity, with practical, tailored advice at the earliest opportunity [51,52].

However, not all HCPs provide advice or specific counselling on PA [53], and less than a quarter do this on a regular basis [54]. In addition, women sometimes feel that HCPs lack the knowledge to answer their questions, and if information is given, it is often unclear or conflicting [27] and does not contain specific advice on the intensity, frequency, and duration of PA [55]. In our own community needs assessments, few of the interviewed Austrian midwives and gynecologists actively talked to pregnant women about PA in pregnancy, and even fewer saw it as their role to try to change the women’s behavior. In SA, while 96.6% of the participating CHWs believed that PA promotion is part of their job, they felt this role was hindered by their own lack of knowledge, training, and confidence to perform the role.

#### 3.1.3. Determinants of Behavior Change

The results from the literature and community needs assessments on HCPs barriers and enablers of PA promotion are presented and sorted by the respective determinants of behavior change. A summary of the findings is provided in Table 1.

##### Knowledge

Pregnant women often receive no PA advice or advice that is limited, conservative, incorrect, or lacking in specificity [45,48,55,56,57], leading them to perceive their midwife’s knowledge on the topic as limited [58]. Indeed, some midwives themselves perceive their knowledge as too limited to give advice and have difficulties with providing recommendations on specific activities [28,59]. This lack of knowledge can be explained by a lack of familiarity with current guidelines and recommendations [53,60,61,62]. In a UK study, as little as 2% correctly identified the current PA in pregnancy guidelines, while nearly 60% reported a high confidence in answering questions relating to it [55]. Indeed, in SA, we encountered similar problems, with only 17% of HCPs aware of the current guidelines [34]. For the CHWs, 69% stated that they were familiar with the PA public health guidelines, and yet 19.7% believed that only vigorous PA is beneficial for health, while 45.9% did not know how to measure intensity. Almost ¼ described this lack of knowledge as a major barrier to giving advice to members of their community.

This lack of knowledge of PA advice and guidelines likely stems from HCPs’ lack of effective training in this area. Leiferman et al. [53] found that 17% of HCPs had never received any training on PA, and out of those who had, 69% rated its quality as fair or poor. Midwives in other studies similarly report that they received little to no formal training on PA during pregnancy [28,59], and very few (4%) reported having access to continued education in this area [55].

Consequently, many HCPs cite common sense, their own experiences, colleagues, or the internet as their sources of information, as opposed to evidence-based resources [55,59,62]. Many midwives are unaware of the professional resources available to them [59], which emphasizes the importance of increasing undergraduate teaching, as well as continued professional development in this area [34,59,61].

Austrian participants of the community needs assessment reported that they had received little to no training on the topic of PA in pregnancy and postpartum during their education and had limited access to post-graduate training on the subject, although most showed interest in future trainings when offered. In SA, only 15.9% of CHWs reported having any formal training in the area of PA, causing a discord between their perceived role and the training received. Current training focused mainly on communicable diseases (HIV/AIDS, tuberculosis) or changes in health policy (immunizations), rather than on PA.

##### Skills

Many HCPs see the task of counselling their patients on PA as a complex challenge [63]. They report both a lack of ability, as well as a lack confidence and self-belief, to adequately advise women [28,53,59], resulting in the feeling that they are unable to induce behavior change in their clients [53]. While they are often aware of the common barriers to becoming active that pregnant women face, addressing those barriers has also been named as a challenge [63].

Similar barriers were observed in the community needs assessment, where only few of the Austrian participants reported that they had received training on facilitating behavior change in pregnant women, while others appeared to have neither the necessary skills nor the confidence for the task. In SA, hardly any CHWs reported having any formal training on behavior change.

##### Social/Professional Role and Identity

Many midwives see giving advice on PA as part of their professional role [28] and acknowledge that they are ideally placed for this role. However, some think that too many aspects of health promotion are placed on them [63,64], and some feel unqualified or question their own suitability as a source of advice and guidance [59]. Some midwives also think it is the women’s general practitioner and obstetric team’s responsibility to counsel women on PA [28], and others reported they have colleagues who think health promotion is unnecessary [64]. Referral to exercise professionals can also be a challenge, where pregnant women are often referred back to their midwife for further advice [59].

In our community needs assessment, it was unclear to the interviewed Austrian midwives who should be responsible for counselling women on the subject, and most identified the mid-pregnancy one-hour consult as an opportunity to discuss PA. In SA, while the majority (96.6%) of CHWs believed that PA promotion is part of their job, a lack of knowledge and training was seen as a major barrier in fulfilling this role.

##### Beliefs about Capabilities

HCPs need confidence to transfer knowledge to patients and to encourage and motivate them in an effective way. However, even though HCPs can have a positive effect on PA levels in pregnant women by means of counselling or education [49], many HCPs feel that they are unable to induce behavior change in their patients [53]. They report feeling frustrated when seeing that their PA promotion efforts were ineffective [63] and report a lack of confidence to advise women [28]. HCPs describe meeting expecting parents’ increasingly high expectations in their professional performance as demanding [63].

Similarly, in the Austrian community needs assessment, HCPs identified overweight and obese women as a risk group, which would especially benefit from improved PA levels. However, they felt that this group was unlikely to change their behavior and that counselling these women would therefore be futile. In SA, many CHWs expressed concern about being ridiculed if they provided the wrong information, especially since pregnant women can readily look up information on the internet or other media sources. They felt that training would equip them to feel confident to provide the right information and answer questions.

##### Beliefs about Consequences

Nearly all midwives and other HCPs acknowledge the importance of PA [28,60]. However, several HCPs report feeling uncomfortable discussing PA with pregnant women and worry about how to communicate effectively without offending and damaging their relationship with them [28,59,63]. Especially when talking to overweight or obese women, PA is seen as a sensitive topic by many caregivers [53]. HCPs think only women who are already active will be interested in specific PA advice in pregnancy [59]. Pregnant women reported that they felt that advice given by their midwife or sports trainer was overly cautious and risk adverse out of a fear that the mother might harm herself or her offspring [27]. A fear of potential litigation was also named a barrier to discussing PA in practice [59].

##### Intentions

While many HCPs are reportedly motivated to promote healthy behavior in pregnant women, they regard it as secondary to their clinical tasks [64] and consequently do not prioritize the promotion of PA or plan to discuss it in daily practice [28,59]. During the interviews in Austria, a large majority of participating midwives and gynecologists also reported that they did not think PA was a topic of priority and consequently did not actively talk to pregnant women about it.

##### Environmental Context and Resources

HCPs report a lack of supportive materials as a barrier to PA counselling, such as educational and motivational leaflets or online tools [59]. Previous formative work in SA has also highlighted the need to provide an information booklet to hand out [65]. Another potential barrier to promoting PA is the lack of awareness of local exercise offerings to which HCPs could refer to [53,59]. In Austria, some HCPs actively referred to local pregnancy-specific activity programs. However, most had very limited knowledge on the programs offered in the area. Several felt a pamphlet to hand out to their patients would be useful, which should also include information about existing local programs. In SA, when discussing what PA training CHWs required, one of the main priorities reported was the provision of specific exercises for pregnant women. They reported the need for specific, standardized information provided on a regular basis. They felt that having a pamphlet to give to pregnant women would also be helpful, as well as a training manual for future reference.

One of the resources most often reported lacking, which therefore hinders the inclusion of PA advice in daily practice, is time [28,53,59,63,64]. A possible explanation for this lack of time is increased pressure at work due to an increase in tasks to perform [63,64], which results in other tasks gaining priority over health promotion [59,64]. A shortage of time was also a widely reported barrier in our community needs assessment, with clinical or other tasks generally being prioritized.

In summary, due to the profound barriers to PA promotion found in HCPs, it is imperative to first address these barriers on the HCP level to create a supportive environment, before focusing on perinatal interventions at the level of the women themselves. The goal of this intervention is therefore to increase the frequency and quality of perinatal PA promotion by HCPs.

### 3.2. Step 2: Formulation of Change Objectives

The desired intervention outcome is specified in more detail by the performance objectives and change objectives. Due to limited resources, the objectives focus on individual level changes, with organizational and community level changes lying outside the scope of this project.

After formulating the performance objectives, the barriers and enablers and their associated behaviors, which were mapped onto TDF domains during the needs assessment, were matched to the performance objectives and reformulated as change objectives in Table 2. Some change objectives were associated with more than one TDF domain. For example, “HCPs see themselves as the right person to promote PA in pregnancy and postpartum” was mapped against the determinants “Social/professional role and identity” and “Beliefs about capabilities”. Barriers were found to represent seven of the 14 domains (Knowledge, Skills, Social/professional role and identity, Beliefs about capabilities, Beliefs about consequences, Intentions, Environmental context, and resources).

### 3.3. Step 3: Selection of Theory-Based Methods and Practical Strategies

In step 3, methods to induce behavior change were selected and translated into practical applications. We selected 14 suitable BCTs linked to the seven TDF domains relevant to our project (Supplementary File S3). Several BCTs apply to more than one TDF domain, for example, the BCT “Problem solving” is linked to the domains “Skills”, “Beliefs about capabilities”, and “Environmental context and resources”.

In Table 3, for each chosen BCT, practical applications are presented. For example, the BCT “Graded tasks” refers to role plays with participants practicing the desired behavior of PA promotion, starting with simple scenarios and progressing towards increasingly complex cases. The practical intervention applications were developed considering their affordability, relevance, and practicality, as well as their acceptability for the intended participants.

### 3.4. Step 4: Intervention Development

The intervention, named “Baby steps”, is designed as an in-person training, combining didactic and interactive education, which has been shown to be effective in changing professional practice [66]. The two-day training will focus on two key points, firstly to equip the participants with all relevant knowledge about PA during and after pregnancy, and secondly to give them tools to transfer this knowledge and to induce behavior change in pregnant women.

The first part of the training will cover the theory of physiological changes during pregnancy and the implications these changes have on PA prescription and the benefits and risks of participating in PA during pregnancy. The PA guidelines and recommendations will be discussed. Participants will receive information on exercise safety and learn to use a standardized, systematic approach to prescribe tailored PA during pregnancy and the postpartum period and when and how to refer to a specialist if needed. Common barriers that women face in becoming active and solutions to overcome them will be addressed.

The second part focuses on knowledge transfer, as well as how to motivate and empower women to change their behavior, while respecting the women’s autonomy. Participants will learn the theory and application of motivational interviewing, a client-centered counselling style aimed at facilitating behavior change [67], which has already been successfully applied to induce behavior change during pregnancy [17]. To apply the learned theory in practice, interactive demonstrations, discussions of case studies, and role plays will be performed. The training in each country will be tailored to fit the different cultures and languages, barriers, and medical background knowledge of South African CHWs and Austrian midwives.

Multiple materials were designed for the intervention. A detailed handbook accompanying the training in each country, containing all contents of the intervention, will be given out to all participants and will include the core elements of the intervention, as well as a list of evidence-based resources, regional specialists, and local programs. As it has been shown that printed educational materials can improve HCPs’ performance [68], an information pamphlet was produced and will be provided to all HCPs to be handed out during the PA promotion. Additionally, a poster to put up in waiting areas will be given out during the training, as it can prompt discussion of the subject. Different versions were produced of all materials, including German and Zulu translations, as well as different culturally relevant pictures and examples. The handbook, pamphlet, and poster can be downloaded from https://baby-steps.international/ (accessed on 27 May 2021).

### 3.5. Step 5: Implementation Plan

Step 5 is concerned with the successful adoption and implementation of the intervention. The program will be adopted and implemented in both countries by the program developers. The goal is to implement the complete intervention in both countries and achieve sustainability through the maintenance of the training at regular intervals. As the program developers are directly involved in the process, we expect a high fidelity and completeness in the implementation of the training.

The training will be offered in a group setting in both countries and will be repeated yearly. In Austria, the training on motivational interviewing will be provided by a psychologist experienced in giving motivational interviewing training. A sports scientist specialized in pregnancy and the postpartum period will provide real-life experiences and examples. Additional support will be given by a midwifery school, including the provision of premises for the training. The training will be credited by the Austrian Midwives Association (Österreichisches Hebammengremium) to increase participation and form part of their continued professional development. It will be embedded in the midwives association’s online system, which offers all credited trainings on the official website and will therefore automatically be accessible to all Austrian midwives. To increase the visibility and thus the recruitment of participants for the training, it will, in addition, be advertised in the Austrian midwifery journal and newsletters. While the current intervention is aimed at qualified midwives, a future goal is to embed the training in their vocational education and to extend the intervention to all healthcare professions associated with pregnancy.

In SA, the training will be facilitated by PA researchers and supported by clinic managers and nurses. It will be run through the public health sector of the organization that employs the CHWs and will therefore be part of their initial recruitment training, as well as a compulsory yearly training. The aim is to demonstrate to the South African government the effectiveness of the training to embed PA promotion into the new national CHWs training curriculum.

### 3.6. Step 6: Evaluation Plan

To assess the intervention effect, the first instalment of the training will serve as a pilot in both countries. The aim of our intervention was to increase the promotion of PA in pregnancy and postpartum by HCPs. To measure the effect of the training on the behavior of HCPs, we developed a survey to be administered immediately before and four weeks after the intervention, assessing how and how often participants promote PA in daily practice (Supplementary File S4). Additional questions will assess whether the intervention had an effect on the various TDF domains and ultimately on the performance and change objectives. The survey is based on a questionnaire by Huijg et al. developed to assess TDF domains [69] and existing questionnaires on PA in pregnancy, aimed at HCPs [28,60]. Additional questions assessing knowledge were based on current PA guidelines.

As part of the process evaluation, we evaluate the reach, recruitment, fidelity, and satisfaction of our training intervention [70]. The reach will measure whether the intervention reached the intended group, which comprises mainly midwives in Austria and CHWs in South Africa, although other HCPs in a position to promote PA around pregnancy will not be excluded. The reach will be assessed by determining how many of the participants of the intervention were part of the intended group. An adequate reach would mean that 100% of participants are HCPs from the target group. As the training in SA will be compulsory for all CHWs, an acceptable recruitment in SA will be reached if at least 70% of all CHWs within the designated region receive the training. In Austria, acceptable recruitment will be reached if, for each training instalment, at least 70% of the total capacity of 20 participants is reached. In addition, we will review whether all strategies on the recruitment and advertising of the training were applied as planned. The fidelity will be assessed by means of the handbook, which contains all core elements of the intervention. Each training session will be compared against the handbook sequence and content. The fidelity will be acceptable if each section was covered during the training. Additional questions were added to the post-intervention effect evaluation survey to assess the HCPs’ satisfaction with the training, including the overall satisfaction with regard to the delivery of the training, training content, and usefulness of the training and materials. Additionally, a focus group is scheduled in both countries at the end of each training session to allow for more in-depth feedback, including barriers and enablers of the effectiveness and implementation of the intervention.

## 4. Discussion and Next Steps

In this paper, we have presented how we used the IM protocol to develop an intervention aimed at healthcare workers in order to equip them with the necessary tools to be able to deliver evidence-based, tailored, high-quality PA promotion to all women during and after pregnancy. The intervention addresses the barriers to PA promotion using theory-based behavior change techniques to achieve the desired behavior in healthcare workers. In the future, a randomized controlled trial will be performed to assess the effectiveness of the intervention.

Our intervention is based on a detailed description of the barriers and enablers found both in the literature and community needs assessments and aims to influence different behavioral determinants of HCPs. It has been shown that the identification and addressing of barriers improves the effectiveness of interventions [71]. The key barriers that we found were a lack of knowledge, skills, and resources, a lack of time and prioritization, and uncertainty about the professional role of HCPs. Similarly, Keyworth et al. [72] found that the barriers of a lack of time, professional role perception, and prioritization were common across different healthcare professions. The authors call for support in identifying opportunities for diverse professional groups to deliver interventions, which is what our research proposes to do. Addressing these barriers is important, as a review investigating the factors influencing primary healthcare professionals’ PA promotion behaviors found that healthcare professionals’ attitudes, intentions, and education on the topic were positively associated with PA promotion [73].

The importance of educating HCPs on the topic of PA was underlined by the findings from our needs assessment, showing that HCPs had limited knowledge about PA in general and specifically during pregnancy. This lack of knowledge is likely caused by the limited availability of undergraduate and continued professional training on the topic, which is also an issue found in other healthcare professions [74]. Our intervention combines education about physical activity with a training session on facilitating behavior change. Training sessions aimed at the acquisition of knowledge and skills are an important enabler for HCPs to deliver individualized and tailored behavior change interventions [51,72], and the ‘making every contact count’ program showed that equipping HCPs with skills to support behavior change, including the promotion of PA, is associated with a significant increase in utilizing these skills in clinical practice [75]. In addition, our intervention is largely based on theoretical approaches and frameworks, which have been shown to improve the effectiveness of the intervention [76], and the use of BCTs ensures that the program is standardized and the results could be replicated in future [36].

Finally, another strength of our study is its development in two economically different countries, expanding the reach of our interventions and making the associated materials more generalizable and relevant to a broader public. The intervention can be adapted and adopted by a variety of professionals in a variety of settings.

Our intervention study has a few limitations. The proposed links between the BCTs and TDF domains used [37] remain hypothetical and are yet to be empirically proven. However, through the systematic and transparent reporting of our intervention development, a replicable intervention is presented, making it possible to identify and investigate individual components, thereby adding to the evidence and providing information for the development of future interventions.

### Next Steps

An RCT is planned to objectively assess the intervention’s effect on HCPs’ behavior, and its effect on women’s PA levels during and after pregnancy. Furthermore, the long-term aim is to integrate the training into HCPs’ vocational education and extend the training to other, relevant professions. Additionally, PA promotion should be addressed on an organizational and community level to increase the reach of the intervention.

## 5. Conclusions

This paper describes how we systematically applied Intervention Mapping to develop an evidence-informed, theory-based intervention, called “Baby steps”, to improve PA promotion by healthcare providers during and after pregnancy and how we plan to implement and evaluate it. The effectiveness of the program will be investigated in the future in a randomized controlled trial. It is clear, from the literature, that intervening to improve PA levels during and after pregnancy is essential for women’s health. This evidence- and theory-based intervention aims at tackling this international problem in various settings.

## Figures and Tables

**Table 1:** Summary of HCP barriers and enablers of PA promotion, sorted by TDF domain.

TDF Domain	Barriers/Enablers
**Knowledge**	Limited knowledge on PA Lack of knowledge of relevant guidelines Limited knowledge of benefits of PA Little training on PA and counselling available
**Skills**	Counselling and addressing women’s barriers to PA seen as complex challenge Lack of skills to induce behavior change
**Social/professional role and identity**	PA promotion seen as part of professional role Unclear which HCP should take on the role Too many health care promotion tasks already placed on HCPs Referral to PA specialists challenging
**Beliefs about capabilities**	Lack of confidence in own counselling skills Counselling of obese or inactive women seen as futile Frustration when PA promotion appears ineffective Increasingly high expectations of expecting parents challenging
**Beliefs about consequences**	Acknowledgement of the importance of PA Afraid of damaging relationship with woman Expectations that women have no interest in PA Fear of litigation Fear of potential negative consequences of PA
**Intentions**	PA not prioritized in daily practice Health promotion seen as secondary to clinical tasks
**Environmental context and resources**	Lack of time in daily practice Lack of supportive materials available Lack of knowledge of local PA programs Lack of knowledge of example exercises

HCP: healthcare provider; PA: physical activity; TDF: Theoretical Domains Framework.

**Table 2:** Performance objectives with mapped change objectives and respective TDF domains.

Performance Objectives (PO)	Change Objectives	TDF Domains
PO1: Healthcare providers are able to explain the physiology and benefits of physical activity during pregnancy and postpartum	HCPs know what constitutes PA	Knowledge
	HCPs are aware of the existing evidence-based guidelines on PA in pregnancy and postpartum and know their content and recommendations	Knowledge
	HCPs acknowledge the importance of PA in pregnancy and postpartum	Beliefs about consequences
	HCPs know about the safety of PA in pregnancy and postpartum and know about (contra-) indications	Knowledge; Social/professional role and identity
	HCPs know where to find evidence-based resources on PA in pregnancy and postpartum	Skills; Environmental context and resources
	HCPs know about the evidence-based benefits of PA in pregnancy and postpartum and its applications as preventive measures and as treatments	Knowledge; Beliefs about consequences
	HCPs are able to obtain continued access to up-to-date training programs for professionals on the topic of PA in pregnancy and postpartum	Knowledge
PO2: Healthcare providers have the necessary skills and are able to provide correct information on recommendations and (contra-) indications of physical activity during pregnancy and postpartum	HCPs can give personalized recommendations according to the F.I.T.T. principle (advise on frequency, intensity, time, and type of PA) and can give recommendations specific to the gestational age or postpartum period	Knowledge; Skills
	HCPs can recognize and correct conflicting information that is given to women on the topic of PA in pregnancy and postpartum	Skills; Beliefs about capabilities
	HCPs know evidence-based methods and skills to promote PA and know methods to motivate and empower women to make changes	Knowledge
	HCPs have the skills and proficiency to promote PA in pregnancy and postpartum in a personalized and evidence-based way	Skills
	HCPs are able to promote PA within a limited time frame	Environmental context and resources
PO3: Healthcare providers transfer their knowledge on physical activity to all women during pregnancy and postpartum	HCPs believe in the importance and effectiveness of counselling on PA in pregnancy and postpartum	Beliefs about capabilities; Beliefs about consequences
	HCPs integrate the topic of PA in daily practice and intend to counsel all women on it during pregnancy and postpartum	Intentions; Goals
	HCPs are confident in their knowledge and their ability to promote PA in pregnancy and postpartum	Beliefs about capabilities
	HCPs see themselves as the right person to promote PA in pregnancy and postpartum	Social/professional role and identity; Beliefs about capabilities
	HCPs see the promotion of PA as their responsibility and as part of their profession	Social/professional role and identity
	HCPs are able to promote PA without damaging their relationship with their clients	Beliefs about consequences
	HCPs use supporting materials, such as folders and websites, to facilitate women in maintaining their activity levels or become physically active in pregnancy or postpartum	Environmental context and resources
PO4: Healthcare providers support, motivate, and empower women to become more physically active during pregnancy and postpartum	HCPs know about existing programs, groups, or professionals of good quality in their area, to which they can refer	Environmental context and resources
	HCPs are confident in their ability to influence women’s motivation and PA levels in pregnancy and postpartum, respecting the women’s autonomy	Beliefs about capabilities
	HCPs are able to determine women’s motivational levels and have the skills to motivate and empower women to do PA in pregnancy and postpartum in an appropriate and evidence-based way, adjusted to the women’s level of motivation, while respecting the women’s autonomy in decision-making	Skills
	HCPs know about common barriers for women, such as social or cultural norms and beliefs about PA, lack of time, or unsafe neighborhoods and can discuss possible solutions	Knowledge; Environmental context and resources
	HCPs can reduce barriers and improve facilitators of women to become physically active or to maintain PA during pregnancy and postpartum	Skills; Environmental context and resources
	HCPs refer women with special needs (i.e., women with GDM or excessive gestational weight gain) to appropriate healthcare professionals for specialized PA prescriptions	Social/professional role and identity

GDM: Gestational Diabetes Mellitus; HCPs: Health care providers; PA: Physical activity; PO: Performance objective; TDF: Theoretical Domains Framework.

**Table 3:** Performance objectives with mapped change objectives and respective TDF domains.

Chosen BCTs	Description BCT (BCTTv1; (35))	Practical Application
Information about health consequences (5.1)	Provide information (e.g., written, verbal, or visual) about health consequences of performing the behavior	Handbook content and presentations during the training intervention will contain information on: Physiological changes during and after pregnancy, guidelines on PA, and health benefits of PA (Contra-) indications and recommendations of PA and exercise safety during and after pregnancy Information about when, how, and where to engage in PA if needed
Information about social and environmental consequences (5.3)	Provide information (e.g., written, verbal, or visual) about social and environmental consequences of performing the behavior	Handbook content and presentations during the intervention will contain information on the (positive) consequences of PA promotion on HCPs’ relationships with women.
Instruction on how to perform the behavior (4.1)	Advise or agree on how to perform the behavior (includes ‘skills training’)	HCPs will receive a theoretical background and instructions on: How to transfer knowledge to their patients and how to influence their behavior through healthy conversations/motivational interviewing/behavior change techniques Exercise prescription recommendations and provision of examples Time management techniques
Feedback on behavior (2.2)	Monitor and provide informative or evaluative feedback on the performance of the behavior (e.g., form, frequency, duration, or intensity)	HCPs will receive feedback on: Their current knowledge and current practice. This will be evaluated by a quiz before the start of the intervention and a repeat quiz at the end of it Their individual performances during role plays from peers and trainers
Behavioral practice/rehearsal (8.1)	Prompt practice or rehearsal of the performance of the behavior one or more times in a context or at a time when the performance may not be necessary in order to increase habit and skill	HCPs will practice listening skills, how to transfer knowledge and motivational interviewing skills as part of role plays, including the practicing of techniques and case studies. Role plays will include individualized exercise prescriptions, providing tailored information, motivational interviewing, discussion of barriers, role plays with a limited time frame, and cases dealing with difficult situations.
Demonstration of the behavior (6.1)	Provide an observable sample of the performance of the behavior, directly in person or indirectly, e.g., via film or pictures, for the person to aspire to or imitate (includes ‘modelling’)	Several real-life and video demonstrations will be performed, including examples of how and when to inform patients about PA and to demonstrate motivational interviewing. Examples will include individualized and tailored information and difficult situations (i.e., a limited time frame or how to deal with incorrect knowledge).
Problem solving (1.2)	Analyze, or prompt the person to analyze, factors influencing the behavior and generate or select strategies that include overcoming barriers and/or increasing facilitators (includes ‘relapse prevention’ and ‘coping planning’)	Barriers and enablers will be addressed: Barriers and enablers to the promotion of PA in HCPs (both the presentation of literature, as well as asking participants for their own experiences) and strategies on how these barriers can be addressed Barriers and enablers of PA in women (the presentation of literature and comparison to participants’ own experiences) and strategies on how these barriers can be addressed, while respecting women’s autonomy
Generalization of target behavior (8.6)	Advise to perform the wanted behavior, which is already performed in a particular situation, in another situation	There will be a brainstorming session, targeting how participants can apply everything they learned in their own daily practice. HCPs will discuss other health-promoting practices or motivational interviewing techniques that they already perform in daily practice, for instance, smoking cessation talks, and try to re-apply it to PA promotion.
Credible source (9.1)	Present verbal or visual communication from a credible source in favor of or against the behavior	As a credible source, experts on PA around pregnancy and motivational interviewing experts will speak at the training. During the intervention and in the handbook, there will be information on other existing credible resources (such as guidelines, etc.) and how and where HCPs can find high-quality, evidence-based additional resources themselves.
Graded tasks (8.7)	Set easy-to-perform tasks, making them increasingly difficult, but achievable, until the behavior is performed	Different role plays with increasing difficulty will be performed by the HCPs, starting with a simple practice of techniques, followed by different case studies with increasing complexity and difficulty.
Discrepancy between current behavior and goal (1.6)	Draw attention to discrepancies between a person’s current behavior (in terms of the form, frequency, duration, or intensity of that behavior) and the person’s previously set outcome goals, behavioral goals, or action plans (goes beyond the self-monitoring of behavior)	Guideline recommendations for women during pregnancy/postpartum will be presented and compared, and the prevalence of women who (do not) reach those recommendations will be discussed. Recommendations that HCPs should promote healthy behavior to all patients will be presented and compared, and the prevalence of HCPs that (do not) talk about PA in practice will be discussed.
Goal setting (outcome) (1.3)	Set or agree on a goal defined in terms of a positive outcome of wanted behavior	HCPs are encouraged to make behavioral resolutions (deciding on how, when, where, and who they will advise on PA).
Adding objects to the environment (12.5)	Add objects to the environment in order to facilitate performance of the behavior	HCPs will receive all information summarized in a handbook, as well as information on local specialists for referral, a leaflet to hand out to their patients, including available classes in the area, and a poster to hang up in their practice.
Prompts and cues (7.1)	Introduce or define environmental or social stimuli with the purpose of prompting or cueing the behavior. The prompt or cue would normally occur at the time or place of the performance	Possible prompts and cues for PA promotion in daily practice will be discussed and practiced in role plays. Prompts and cues can be certain openings during a talk with a patient, but could also be a memo in a patient file reminding HCPs to discuss PA.

BCT: ehavior Change Technique; BCTTv1: Behavior Change Technique Taxonomy Version 1; HCPs: Health care providers; PA: Physical activity; TDF: Theoretical Domains Framework.

## Data Availability

The data presented in this study are available on reasonable request from the corresponding author.

## Supplementary Materials

The following are available online at https://www.mdpi.com/article/10.3390/ijerph18115869/s1, File S1: TiDieR Checklist, File S2: Needs assessment survey for community health workers, File S3: Table describing the chosen BCTs linked to TDF domains, File S4: Survey on Physical Activity in Pregnancy and Postpartum for Healthcare Providers.

## References

[1] Brown W. (2002). The benefits of physical activity during pregnancy. J. Sci. Med. Sport.

[2] Mudd L.M., Owe K.M., Mottola M.F., Pivarnik J.M. (2013). Health benefits of physical activity during pregnancy: An international perspective. Med. Sci. Sports Exerc..

[3] Schlüssel M.M., De Souza E.B., Reichenheim M.E., Kac G. (2008). Physical activity during pregnancy and maternal-child health outcomes: A systematic literature review. Cadernos Saúde Pública.

[4] Aune D., Sen A., Henriksen T., Saugstad O.D., Tonstad S. (2016). Physical activity and the risk of gestational diabetes mellitus: A systematic review and dose–response meta-analysis of epidemiological studies. Eur. J. Epidemiol..

[5] Davenport M.H., Ruchat S.-M., Poitras V.J., Garcia A.J., Gray C., Barrowman N., Skow R.J., Meah V.L., Riske L., Sobierajski F. (2018). Prenatal exercise for the prevention of gestational diabetes mellitus and hypertensive disorders of pregnancy: A systematic review and meta-analysis. Br. J. Sports Med..

[6] Ruchat S.-M., Mottola M.F., Skow R.J., Nagpal T.S., Meah V.L., James M., Riske L., Sobierajski F., Kathol A.J., Marchand A.-A. (2018). Effectiveness of exercise interventions in the prevention of excessive gestational weight gain and postpartum weight retention: A systematic review and meta-analysis. Br. J. Sports Med..

[7] Artal R. (2016). Exercise in Pregnancy: Guidelines. Clin. Obstet. Gynecol..

[8] Mottola M.F., Davenport M.H., Ruchat S.M., Davies G.A., Poitras V.J., Gray C.E., Garcia A.J., Barrowman N., Adamo K.B., Duggan M. (2018). 2019 Canadian guideline for physical activity throughout pregnancy. Br. J. Sports Med..

[9] American College of Obstetricians and Gynecologists (2020). Physical Activity and Exercise during Pregnancy and the Postpartum Period: ACOG Committee Opinion, Number 804. Obstet. Gynecol..

[10] Borodulin K., Evenson K.R., Herring A.H. (2009). Physical activity patterns during pregnancy through postpartum. BMC Women’s Health.

[11] Borodulin K.M., Evenson K.R., Wen F., Herring A.H., Benson A.M. (2008). Physical activity patterns during pregnancy. Med. Sci. Sports Exerc..

[12] Evenson K.R., Wen F. (2010). National trends in self-reported physical activity and sedentary behaviors among pregnant women: NHANES 1999–2006. Prev. Med..

[13] Hesketh K.R., Evenson K.R. (2016). Prevalence of U.S. Pregnant Women Meeting 2015 ACOG Physical Activity Guidelines. Am. J. Prev. Med..

[14] Poudevigne M.S., O’Connor P.J. (2006). A Review of Physical Activity Patterns in Pregnant Women and Their Relationship to Psychological Health. Sports Med..

[15] Watson E.D., Brage S., White T., Westgate K., Norris S.A., Van Poppel M.N.M., Micklesfield L.K. (2018). The Influence of Objectively Measured Physical Activity During Pregnancy on Maternal and Birth Outcomes in Urban Black South African Women. Matern. Child Health J..

[16] Watson E.D., Van Poppel M.N.M., Jones R.A., Norris A.S., Micklesfield L.K. (2017). Are South African Mothers Moving? Patterns and Correlates of Physical Activity and Sedentary Behavior in Pregnant Black South African Women. J. Phys. Act. Health.

[17] Simmons D., Devlieger R., Van Assche A., Jans G., Galjaard S., Corcoy R., Adelantado J.M., Dunne F., Desoye G., Harreiter J. (2016). Effect of Physical Activity and/or Healthy Eating on GDM Risk: The DALI Lifestyle Study. J. Clin. Endocrinol. Metab..

[18] Hesketh K.R., Evenson K.R., Stroo M., Clancy S.M., Østbye T., Benjamin-Neelon S.E. (2018). Physical activity and sedentary behavior during pregnancy and postpartum, measured using hip and wrist-worn accelerometers. Prev. Med. Rep..

[19] Pereira M.A., Rifas-Shiman S.L., Kleinman K.P., Rich-Edwards J.W., Peterson K.E., Gillman M.W. (2007). Predictors of Change in Physical Activity During and After Pregnancy: Project Viva. Am. J. Prev. Med..

[20] Thompson E.L., Vamos C.A., Daley E.M. (2017). Physical activity during pregnancy and the role of theory in promoting positive behavior change: A systematic review. J. Sport Health Sci..

[21] Harrison A.L., Taylor N.F., Shields N., Frawley H.C. (2018). Attitudes, barriers and enablers to physical activity in pregnant women: A systematic review. J. Physiother..

[22] Jelsma J.G.M., van Leeuwen K., Oostdam N., Bunn C., Simmons D., Desoye G., Corcoy R., Adelantado J.M., Kautzky-Willer A., Harreiter J. (2016). Beliefs, Barriers, and Preferences of European Overweight Women to Adopt a Healthier Lifestyle in Pregnancy to Minimize Risk of Developing Gestational Diabetes Mellitus: An Explorative Study. J. Pregnancy.

[23] Evenson K.R., Aytur S.A., Borodulin K. (2009). Physical activity beliefs, barriers, and enablers among postpartum women. J. Womens Health.

[24] Watson E.D., Norris S.A., Draper C.E., Jones R.A., van Poppel M.N.M., Micklesfield L.K. (2016). “Just because you’re pregnant, doesn’t mean you’re sick!” A qualitative study of beliefs regarding physical activity in black South African women. BMC Pregnancy Childbirth.

[25] Coll C., Domingues M., Santos I., Matijasevich A., Horta B.L., Hallal P.C. (2016). Changes in Leisure-Time Physical Activity from the Prepregnancy to the Postpartum Period: 2004 Pelotas (Brazil) Birth Cohort Study. J. Phys. Act. Health.

[26] Clarke P.E., Gross H. (2004). Women’s behaviour, beliefs and information sources about physical exercise in pregnancy. Midwifery.

[27] Findley A., Smith D.M., Hesketh K., Keyworth C. (2020). Exploring womens’ experiences and decision making about physical activity during pregnancy and following birth: A qualitative study. BMC Pregnancy Childbirth.

[28] McParlin C., Bell R., Robson S.C., Muirhead C., Araújo-Soares V. (2017). What helps or hinders midwives to implement physical activity guidelines for obese pregnant women? A questionnaire survey using the Theoretical Domains Framework. Midwifery.

[29] Bartholomew L.K., Parcel G.S., Kok G., Gottlieb N.H., Schaalma H.C., Markham C.C., Tyrrell S.C., Shegog R.C., Fernández M.C., Mullen P.D.C. (2006). Planning Health Promotion Programs: An Intervention Mapping Approach.

[30] Craig P., Dieppe P., Macintyre S., Michie S., Nazareth I., Petticrew M. (2008). Medical Research Council: Developing and evaluating complex interventions: New guidance. Br. Med. J..

[31] Taylor N., Conner M., Lawton R. (2012). The impact of theory on the effectiveness of worksite physical activity interventions: A meta-analysis and meta-regression. Health Psychol. Rev..

[32] Bartholomew L.K., Parcel G.S., Kok G., Gottlieb N.H., Fernández M.E. (2011). Planning Health Promotion Programs: An Intervention Mapping Approach.

[33] Hoffmann T.C., Glasziou P.P., Boutron I., Milne R., Perera R., Moher D., Altman D.G., Barbour V., Macdonald H., Johnston M. (2014). Better reporting of interventions: Template for intervention description and replication (TIDieR) checklist and guide. BMJ.

[34] Watson E.D., Oddie B., Constantinou D. (2015). Exercise during pregnancy: Knowledge and beliefs of medical practitioners in South Africa: A survey study. BMC Pregnancy Childbirth.

[35] Cane J., O’Connor D., Michie S. (2012). Validation of the theoretical domains framework for use in behaviour change and implementation research. Implement. Sci..

[36] Michie S., Richardson M., Johnston M., Abraham C., Francis J., Hardeman W., Eccles M.P., Cane J., Wood C.E. (2013). The Behavior Change Technique Taxonomy (v1) of 93 Hierarchically Clustered Techniques: Building an International Consensus for the Reporting of Behavior Change Interventions. Ann. Behav. Med..

[37] Johnston M., Carey R.N., Bohlen L.E.C., Johnston D.W., Rothman A.J., De Bruin M., Kelly M.P., Groarke H., Michie S. (2020). Development of an online tool for linking behavior change techniques and mechanisms of action based on triangulation of findings from literature synthesis and expert consensus). Transl. Behav. Med..

[38] Bundesminister für soziale Sicherheit und Generationen (2002). Verordnung des Bundesministers für soziale Sicherheit und Generationen über die Festlegung eines Mutter-Kind-Pass-Untersuchungsprogrammes, die Voraussetzungen zur Weitergewährung des Kinderbetreuungsgeldes in voller Höhe sowie über den Mutter-Kind-Pass Austria. https://www.ris.bka.gv.at/GeltendeFassung/Bundesnormen/20001694/MuKiPassV%2CFassungvom.pdf.

[39] Wilford A., Phakathi S., Haskins L., Jama N.A., Mntambo N., Horwood C. (2018). Exploring the care provided to mothers and children by community health workers in South Africa: Missed opportunities to provide comprehensive care. BMC Public Health.

[40] Le Roux K., Le Roux I.M., Mbewu N., Davis E. (2015). The role of community health workers in the re-engineering of primary health care in rural eastern cape. South Afr. Fam. Pract..

[41] Daviaud E., Nkonki L., Ijumba P., Doherty T., Lawn E.J., Owen H., Jackson D., Tomlinson M. (2017). South-Africa (Goodstart III) trial: Community-based maternal and newborn care economic analysis. Health Policy Plan..

[42] Bosire E.N., Ware L.J., Draper C.E., Amato B., Kapueja L., Lye S., Norris S.A. (2021). Young women’s perceptions of life in urban South Africa: Contextualising the preconception knowledge gap. Afr. J. Reprod. Health.

[43] Coll C.V., Domingues M.R., Gonçalves H., Bertoldi A.D. (2017). Perceived barriers to leisure-time physical activity during pregnancy: A literature review of quantitative and qualitative evidence. J. Sci. Med. Sport.

[44] Alvis M.L., Morris C.E., Garrard T.L., Hughes A.G., Hunt L., Koester M.M., Yocum I.C., Tinius R.A. (2019). Educational Brochures Influence Beliefs and Knowledge Regarding Exercise during Pregnancy: A Pilot Study. Int. J. Exerc. Sci..

[45] Stengel M.R., Kraschnewski J.L., Hwang S.W., Kjerulff K.H., Chuang C.H. (2012). “What My Doctor Didn’t Tell Me”: Examining Health Care Provider Advice to Overweight and Obese Pregnant Women on Gestational Weight Gain and Physical Activity. Women’s Health Issues.

[46] Santo E.C., Forbes P.W., Oken E., Belfort M.B. (2017). Determinants of physical activity frequency and provider advice during pregnancy. BMC Pregnancy Childbirth.

[47] Van der Pligt P., Olander E.K., Ball K., Crawford D., Hesketh K.D., Teychenne M., Campbell K. (2016). Maternal dietary intake and physical activity habits during the postpartum period: Associations with clinician advice in a sample of Australian first time mothers. BMC Pregnancy Childbirth.

[48] Ferrari R.M., Siega-Riz A.M., Evenson K.R., Moos M.-K., Carrier K.S. (2013). A qualitative study of women’s perceptions of provider advice about diet and physical activity during pregnancy. Patient Educ. Couns..

[49] Currie S., Sinclair M., Murphy M.H., Madden E., Dunwoody L., Liddle D. (2013). Reducing the Decline in Physical Activity during Pregnancy: A Systematic Review of Behaviour Change Interventions. PLoS ONE.

[50] Pearce E.E., Evenson K.R., Downs D.S., Steckler A. (2013). Strategies to Promote Physical Activity during Pregnancy. Am. J. Lifestyle Med..

[51] National Institute for Health and Care Excellence (NICE) (2010). Weight Management before, during and after Pregnancy (PH27).

[52] World Health Organisation (2016). WHO Recommendation on Antenatal Care for Positive Pregnancy Experience.

[53] Leiferman J., Gutilla M., Paulson J., Pivarnik J. (2012). Antenatal physical activity counseling among healthcare providers. Open J. Obstet. Gynecol..

[54] McGee L.D., Cignetti A.C., Sutton A., Harper L., Dubose C., Gould S. (2018). Exercise During Pregnancy: Obstetricians’ Beliefs and Recommendations Compared to American Congress of Obstetricians and Gynecologists’ 2015 Guidelines. Cureus.

[55] Hopkinson Y., Hill D.M., Fellows L., Fryer S. (2018). Midwives understanding of physical activity guidelines during pregnancy. Midwifery.

[56] Hayman M., Reaburn P., Alley S., Cannon S., Short C. (2020). What exercise advice are women receiving from their healthcare practitioners during pregnancy?. Women Birth.

[57] Entin P.L., Munhall K.M. (2006). Recommendations regarding exercise during pregnancy made by private/small group practice obstetricians in the USA. J. Sports Sci. Med..

[58] Flannery C., McHugh S., Anaba A.E., Clifford E., O’Riordan M., Kenny L.C., McAuliffe F.M., Kearney P.M., Byrne M. (2018). Enablers and barriers to physical activity in overweight and obese pregnant women: An analysis informed by the theoretical domains framework and COM-B model. BMC Pregnancy Childbirth.

[59] De Vivo M., Mills H. (2019). “They turn to you first for everything”: Insights into midwives’ perspectives of providing physical activity advice and guidance to pregnant women. BMC Pregnancy Childbirth.

[60] Bauer P.W., Broman C.L., Pivarnik J.M. (2010). Exercise and Pregnancy Knowledge among Healthcare Providers. J. Women’s Health.

[61] Evenson K.R., Pompeii L.A. (2010). Obstetrician Practice Patterns and Recommendations for Physical Activity during Pregnancy. J. Women’s Health.

[62] Crampton J.S., O’Brien S., Heathcote K. (2018). Recreational exercise during pregnancy: Attitudes and beliefs of midwives and physiotherapists. Br. J. Midwifery.

[63] Lindqvist M., Mogren I., Eurenius E., Edvardsson K., Persson M. (2014). “An on-going individual adjustment”: A qualitative study of midwives’ experiences counselling pregnant women on physical activity in Sweden. BMC Pregnancy Childbirth.

[64] McLellan J.M., O’Carroll R.E., Cheyne H., Dombrowski S.U. (2019). Investigating midwives’ barriers and facilitators to multiple health promotion practice behaviours: A qualitative study using the theoretical domains framework. Implement. Sci..

[65] Muzigaba M., Kolbe-Alexander T.L., Wong F. (2014). The Perceived Role and Influencers of Physical Activity among Pregnant Women From Low Socioeconomic Status Communities in South Africa. J. Phys. Act. Health.

[66] Forsetlund L., Bjørndal A., Rashidian A., Jamtvedt G., O’Brien M.A., Wolf F.M., Davis D., Odgaard-Jensen J., Oxman A.D. (2009). Continuing education meetings and workshops: Effects on professional practice and health care outcomes. Cochrane Database Syst. Rev..

[67] Miller W.R., Rollnick S. (1991). Motivational Interviewing: Preparing People to Change Addictive Behaviours.

[68] Giguère A., Zomahoun H.T.V., Carmichael P.-H., Uwizeye C.B., Légaré F., Grimshaw J., Gagnon M.-P., Auguste D.U., Massougbodji J. (2020). Printed educational materials: Effects on professional practice and healthcare outcomes. Cochrane Database Syst. Rev..

[69] Huijg J.M., A Gebhardt W., Crone M.R., Dusseldorp E., Presseau J. (2014). Discriminant content validity of a theoretical domains framework questionnaire for use in implementation research. Implement. Sci..

[70] Linnan L., Steckler A. (2002). Process evaluation for public health interventions and research: An overview. Process Evaluation for Public Health Interventions and Research.

[71] Johnson M.J., May C.R. (2015). Promoting professional behaviour change in healthcare: What interventions work, and why? A theory-led overview of systematic reviews. BMJ Open.

[72] Keyworth C., Epton T., Goldthorpe J., Calam R., Armitage C.J. (2020). Delivering Opportunistic Behavior Change Interventions: A Systematic Review of Systematic Reviews. Prev. Sci..

[73] Huijg J.M., Gebhardt W.A., Verheijden M.W., Van Der Zouwe N., De Vries J.D., Middelkoop B.J.C., Crone M.R. (2014). Factors Influencing Primary Health Care Professionals’ Physical Activity Promotion Behaviors: A Systematic Review. Int. J. Behav. Med..

[74] Weiler R., Chew S., Coombs N., Hamer M., Stamatakis E. (2012). Physical activity education in the undergraduate curricula of all UK medical schools. Are tomorrow’s doctors equipped to follow clinical guidelines?. Br. J. Sports Med..

[75] Lawrence W., Black C., Tinati T., Cradock S., Begum R., Jarman M., Pease A., Margetts B., Davies J., Inskip H. (2016). ‘Making every contact count’: Evaluation of the impact of an intervention to traiAn health and social care practitioners in skills to support health behaviour change. J. Health Psychol..

[76] Brug J., Oenema A., Ferreira I. (2005). Theory, evidence and Intervention Mapping to improve behavior nutrition and physical activity interventions. Int. J. Behav. Nutr. Phys. Act..

