# Supplementary material for: Baby Steps: Using Intervention Mapping to Develop a Sustainable Perinatal Physical Activity Healthcare Intervention
Source: Int J Environ Res Public Health. 2021 May 30;18(11):5869. doi: 10.3390/ijerph18115869 (PMC8198094; doi:10.3390/ijerph18115869)
Supplement: Supplementary file 1 [file ijerph-18-05869-s001.zip › Supp_2_CHWsurvey.pdf]

Anna M. Dieberger; Mireille N. M. van Poppel; Estelle D. Watson

Baby steps: Using Intervention Mapping to develop a sustainable perinatal physical activity healthcare intervention

Supplementary File 2: Needs assessment survey for community health workers

Survey CHWs

ID Code	

**Please tick the response that most closely fits your demographics. If the choices given do not fit your situation, please feel free to write in one that does.**					
1. How many years have you been working as a community health worker?	☐ <2 years	☐ 2-5 years	☐ 5-10 years	☐ 10-20 years	☐>20
2. What is your gender?	☐ Male	☐ Female			
3. What is your race?	☐ Caucasian	☐African	☐Indian	☐Coloured	Other___________
4. What is your age?					
5. On a scale of 1-5 how would you rate your skills and knowledge needed to be a community health worker?	☐ 1<br>Not highly skilled	☐2	☐3<br>Somewhat skilled	☐4	☐5<br>Highly skilled
Please describe how you would rate your skills in the following roles	Beginner	Intermediate	Advanced		
6. Communication skills	☐	☐	☐		
7. Interpersonal skills	☐	☐	☐		
8. Advocating for individual and community needs	☐	☐	☐		
9. Organizational skills	☐	☐	☐		
10. Assuring that people get the services they need	☐	☐	☐		
11. Broad knowledge about community and health issues	☐	☐	☐		
12. Providing direct services	☐	☐	☐		
13. Teaching skills	☐	☐	☐		
14. Providing culturally appropriate health education	☐	☐	☐		
15. Informal counselling and social support	☐	☐	☐		
16. Capacity building skills	☐	☐	☐		
17. Cultural mediation between communities and health and services	☐	☐	☐		

**For the following questions, physical Activity refers to any activity that you do from a low intensity level, such as walking, to a high intensity level, such as playing a competitive sport.**					
18. Are you familiar with the guidelines on how much physical activity you should do to be healthy?	☐ Yes	☐ No			
19. How many ***minutes*** of physical activity should one do per week to gain health benefits?	Minutes / week				
20. At what ***intensity*** should the activity be done?	☐ Low intensity	☐ Moderate intensity	☐ Heavy intensity		
21. How would you measure intensity whilst exercising?					
22. Provide an example of an activity for each intensity level					
23. How many days per week should aerobic exercise be performed? (exercise that increases your heart and breathing rate such as walking, cycling)					
24. How many days per week should strength/resistance exercise be performed? (such as using weights or doing squats)					
25. How many days per week should stretching exercises be performed?					

Please tick the response that most closely fits your feelings toward the statement given. If examples are requested, please write all those that you feel apply in the space provided.

26. Physical activity / exercising is beneficial for your health.

☐ Strongly agree ☐ Agree ☐ Disagree ☐ Strongly disagree

27. What do you feel are the main benefits of physical activity?

☐ Decreased risk of high blood pressure
☐ Decreases risk of Diabetes
☐ Decreased risk of high cholesterol
☐ Decreased risk of heart attack
☐ Decreased risk of stroke
☐ Decreased risk of breast cancer
☐ Improved mood
☐ Weight management
☐ Better posture
☐ Increased fitness levels
☐ Increased strength levels
☐ Improved self-image
☐ Better sleeping patterns
☐ Decreased risk of depression
☐ Improved ability to move
☐ Better quality of life
☐ Other:________________________________

28. Any amount of physical activity is beneficial to health.

☐ Strongly agree ☐ Agree ☐ Disagree ☐ Strongly disagree

29. Only vigorous/heavy activity is beneficial to health.

☐ Strongly agree ☐ Agree ☐ Disagree ☐ Strongly disagree

30. Promoting physical activity is an important part of my job as a community health worker.

☐ Strongly agree ☐ Agree ☐ Disagree ☐ Strongly disagree

31. I regularly advise my community about the benefits of physical activity

☐ Strongly agree ☐ Agree ☐ Disagree ☐ Strongly disagree

32. How do you do this (provide examples)

____
____
____
____

33. I have sufficient knowledge to advice people about physical activity.

☐ Strongly agree ☐ Agree ☐ Disagree ☐ Strongly disagree

34. I believe that I can help the community to increase their physical activity levels.

☐ Strongly agree ☐ Agree ☐ Disagree ☐ Strongly disagree

35. Do you recommend exercise / physical activity to your patients?

☐ Yes ☐ No -- If no, please skip to question 37.

a. What percentage of patients do you recommend physical activity /exercise?

☐ 25% ☐ 50% ☐ 75% ☐ All my patients

b. For what reasons would you NOT recommend physical activity / exercise?

c. If yes, what types of exercise do you recommend for people in your community? (Please tick all that apply; feel free to add others.)

☐ Walking ☐ Running ☐ Swimming ☐ Cycling ☐ Aerobics

Other ____

____

36. What percentage of your patients in the community asks about physical activity?

☐ None ☐ 25% ☐ 50% ☐ 75% ☐ All of them

37. Are you aware of any exercise classes or physical activity programmes in your area?
☐ Yes ☐ No

If yes, please describe them: ____

____

____

If no, please skip question (a) below.

a. If yes, do you recommend people to go to any of these opportunities?

☐ Never ☐ Seldom ☐ Often ☐ Always

Please provide us with information regarding your training needs and experiences regarding physical activity

38. Have you ever received formal training in PA/exercise?
☐ Yes ☐ No

a. If yes, how long was this training?

____

____

b. Where did this training take place?

____

____

c. Who provided this training?

____

____

39. How interested would you be in receiving formal training on physical activity / exercise

☐ Not interested ☐ Slightly interested ☐ Interested ☐ Very interested

40. How would you like to receive training in PA/exercise?

___

___

41. What type of things would you like to learn about in terms of PA/exercise?

___

___

42. How likely would you be to apply training in PA/exercise in your work with patients in the community setting?

☐ Not likely ☐ Likely ☐ Extremely likely
