# Supplementary material for: Baby Steps: Using Intervention Mapping to Develop a Sustainable Perinatal Physical Activity Healthcare Intervention
Source: Int J Environ Res Public Health. 2021 May 30;18(11):5869. doi: 10.3390/ijerph18115869 (PMC8198094; doi:10.3390/ijerph18115869)
Supplement: Supplementary file 1 [file ijerph-18-05869-s001.zip › Supp_3_Table.pdf]

Anna M. Dieberger; Mireille N. M. van Poppel; Estelle D. Watson

Baby steps: Using Intervention Mapping to develop a sustainable perinatal physical activity healthcare intervention

Supplementary File 3: Table describing chosen BCTs linked to TDF domains

Supplementary File 3: Chosen BCTs linked to TDF domains

TDF Domain	BCTs
Knowledge	Information about health consequences (5.1)*<br>Information about social and environmental consequences (5.3)*<br>Instruction on how to perform the behaviour (4.1)*<br>Feedback on behaviour (2.2)
Skills	Behavioural practice / rehearsal (8.1)*<br>Demonstration of the behaviour (6.1)*<br>Instruction on how to perform the behaviour (4.1)*<br>Problem solving (1.2)*<br>Generalization of target behaviour (8.6)
Social / professional role and identity	Credible source (9.1)
Beliefs about capabilities	Graded tasks (8.7)<br>Demonstration of the behaviour (6.1)*<br>Problem solving (1.2)*<br>Behavioural practice / rehearsal (8.1)*
Beliefs about consequences	Information about health consequences (5.1)*<br>Information about social and environmental consequences (5.3)*
Intentions / Goals[§]	Information about health consequences (5.1)*<br>Discrepancy between current behaviour and goal (1.6)<br>Goal setting (outcome) (1.3)
Environmental context and resources	Adding objects to the environment (12.5)<br>Problem solving (1.2)*<br>Prompts & cues (7.1)

BCT: Behaviour Change Technique; TDF: Theoretical Domains Framework. *BCT applies to more than one TDF domain. [§]Domains 'Intentions' and 'Goals' combined into one domain ("Intentions").

Based on Johnston M, Carey RN, Connell Bohlen LE, Johnston DW, Rothman AJ, de Bruin M, et al. Development of an online tool for linking behavior change techniques and mechanisms of action based on triangulation of findings from literature synthesis and expert consensus. Transl Behav Med. 2020
