# Supplementary material for: Baby Steps: Using Intervention Mapping to Develop a Sustainable Perinatal Physical Activity Healthcare Intervention
Source: Int J Environ Res Public Health. 2021 May 30;18(11):5869. doi: 10.3390/ijerph18115869 (PMC8198094; doi:10.3390/ijerph18115869)

Anna M. Dieberger; Mireille N. M. van Poppel; Estelle D. Watson

Baby steps: Using Intervention Mapping to develop a sustainable perinatal physical activity healthcare intervention

Supplementary File 4: Survey on Physical Activity in Pregnancy and Postpartum for Healthcare Providers

Survey on Physical Activity in Pregnancy and Postpartum for Healthcare Providers

Thank you for taking the time to complete this survey. It will help us to improve the training and resources that are needed to help healthcare providers to inform and support women to do sufficient physical activity in pregnancy and the postpartum period.

By **physical activity** we mean **any bodily movement that expends energy**. This includes not only sport but also walking to the supermarket, taking the stairs or cleaning.

All **answers** will be analysed **anonymously**. There are no right or wrong answers, as we are interested in your views, beliefs and opinions.

Personal Information

1. **What is your profession?**

☐	☐	☐	☐	☐
Midwife	General practitioner	Obstetrician	Community Health Worker	Other: ___________

2. **During which stage of pregnancy do you usually see patients?** ***(multiple answers possible)***

☐	☐	☐	☐	☐	☐
Before pregnancy	1st trimester	2nd trimester	3rd trimester	1st week after birth	> 1st week after birth

3. **How physically active would you describe yourself?**

Very inactive / sedentary	1	2	3	4	5	6	7	8	9	10	Very active

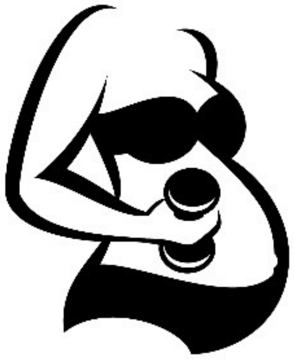

		Always	Usually	Sometimes	Rarely	Never
**Current behaviour**						
4.	I advise women to be physically active during pregnancy.	☐	☐	☐	☐	☐
5.	I advise women to be physically active in the postpartum period.	☐	☐	☐	☐	☐
6.	I inform women about the health benefits of physical activity during and after pregnancy.	☐	☐	☐	☐	☐
7.	I initiate talking about physical activity with all pregnant women.	☐	☐	☐	☐	☐
8.	I provide specific recommendations on type, frequency, time and intensity of exercises to women during and after pregnancy.	☐	☐	☐	☐	☐
9.	I recommend local exercise classes and physical activity programmes to pregnant and postpartum women.	☐	☐	☐	☐	☐
10.	I determine a woman's motivational level to change her behaviour and adapt my counselling on physical activity accordingly.	☐	☐	☐	☐	☐

		Strongly agree	Agree	Neither agree nor disagree	Disagree	Strongly disagree
**Knowledge**						
11.	I am aware of the existence of national guidelines regarding physical activity during pregnancy.	☐	☐	☐	☐	☐
12.	Pregnant women should participate in 150 minutes of moderate physical activity per week or more.	☐	☐	☐	☐	☐
13.	Women should do pelvic floor strengthening exercises (i.e. Kegel exercises) during and after pregnancy.	☐	☐	☐	☐	☐
14.	Exercising during pregnancy is beneficial.	☐	☐	☐	☐	☐
15.	Pregnant women who are regular exercisers should be encouraged to continue an exercise programme throughout pregnancy.	☐	☐	☐	☐	☐
16.	I have sufficient knowledge to give advice about physical activity.	☐	☐	☐	☐	☐
17.	If women have not exercised prior to pregnancy, they should not attempt to start during pregnancy.	☐	☐	☐	☐	☐
18.	Experts don't really know if physical activity is beneficial or harmful for obese pregnant women.	☐	☐	☐	☐	☐

		Strongly agree	Agree	Neither agree nor disagree	Disagree	Strongly disagree
**Attitude**						
19.	I am keen to implement the guidelines regarding physical activity for pregnant and postpartum women.	☐	☐	☐	☐	☐
20.	I feel uncomfortable talking to women who are inactive / obese about their physical activity levels.	☐	☐	☐	☐	☐
21.	I have more important things to discuss with pregnant women during prenatal appointments than their physical activity levels.	☐	☐	☐	☐	☐
**Skills**						
22.	If I encounter a problem when discussing physical activity with pregnant or postpartum women I know how to solve it.	☐	☐	☐	☐	☐
23.	I am able to implement the recommendations regarding physical activity and pregnant / postpartum women.	☐	☐	☐	☐	☐
24.	I have a clear plan of how to address physical activity issues with pregnant women.	☐	☐	☐	☐	☐
**Beliefs in Capabilities**						
25.	I feel competent answering questions about physical activity in pregnancy and the postpartum period.	☐	☐	☐	☐	☐
26.	I believe that I can help women increase their physical activity levels.	☐	☐	☐	☐	☐
27.	I feel optimistic that if I give physical activity advice to women, they will follow it.	☐	☐	☐	☐	☐
**Beliefs in Consequences**						
28.	The possible harmful effects of moderate intensity exercise on the foetus are minimal if not non-existent.	☐	☐	☐	☐	☐
29.	I worry about making pregnant women anxious by advising and encouraging them to be more active.	☐	☐	☐	☐	☐
30.	Inactive / obese pregnant women will feel guilty if I discuss physical activity with them and they find it difficult to be more active.	☐	☐	☐	☐	☐
31.	I worry about damaging my relationship with the women when advising to be more active.	☐	☐	☐	☐	☐

		Strongly agree	Agree	Neither agree nor disagree	Disagree	Strongly disagree
32.	Advising women to be more physically active during pregnancy may put their baby at risk of harm.	☐	☐	☐	☐	☐
**Social / Professional role and identity**						
33.	Advising patients on exercise during and after pregnancy is not a major component of prenatal care.	☐	☐	☐	☐	☐
34.	Pregnant and postpartum women expect me to discuss physical activity with them.	☐	☐	☐	☐	☐
35.	It is part of my role as a healthcare professional to advise women about the appropriate type and amount of physical activity they should do during and after pregnancy.	☐	☐	☐	☐	☐
36.	It is part of my role to discuss physical activity during routine care with pregnant / postpartum women.	☐	☐	☐	☐	☐
37.	My colleagues do not discuss physical activity routinely with pregnant women.	☐	☐	☐	☐	☐
**Environmental context and resources**						
38.	I am usually too busy to discuss physical activity with pregnant /postpartum women.	☐	☐	☐	☐	☐
39.	I am trained to give pregnant women appropriate advice regarding physical activity during and after pregnancy.	☐	☐	☐	☐	☐
40.	I received formal training on how to motivate and inform pregnant / postpartum women on public health messages.	☐	☐	☐	☐	☐
41.	If I have any questions regarding physical activity in pregnant women there is no one readily available to give me the support and answers I need.	☐	☐	☐	☐	☐
42.	I know resources where I can find evidence-based answers to questions regarding physical activity during and after pregnancy.	☐	☐	☐	☐	☐
43.	I know about local exercise classes and physical activity programmes for women during and after pregnancy.	☐	☐	☐	☐	☐

Satisfaction with training

Please let us know how satisfied you are with the training you received these past 2 days and the provided materials.

		Very much	Somewhat	Undecided	Not really	Not at all
1.	How satisfied are you with the training overall?	☐	☐	☐	☐	☐
2.	How satisfied are you with the content of the training?	☐	☐	☐	☐	☐
3.	How useful did you find the training?	☐	☐	☐	☐	☐
4.	How useful do you find the provided materials?	☐	☐	☐	☐	☐

Comments and suggestions

____

____

____

____

____

THANK YOU!

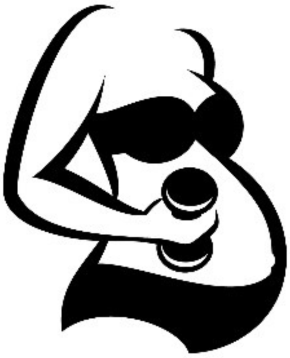

Supplement: Supplementary file 1 [file ijerph-18-05869-s001.zip › Supp_4_Survey.pdf]
